# Physical Vein Models to Quantify the Flow Performance of Sclerosing Foams

**DOI:** 10.3389/fbioe.2019.00109

**Published:** 2019-05-21

**Authors:** Elisabetta Bottaro, Jemma Paterson, Xunli Zhang, Martyn Hill, Venisha A. Patel, Stephen A. Jones, Andrew L. Lewis, Timothy M. Millar, Dario Carugo

**Affiliations:** ^1^Faculty of Engineering and Physical Sciences, University of Southampton, Southampton, United Kingdom; ^2^Faculty of Medicine, University of Southampton, Southampton, United Kingdom; ^3^Institute for Life Sciences (IfLS), University of Southampton, Southampton, United Kingdom; ^4^Biocompatibles UK Ltd. (a BTG group company), Camberley, United Kingdom

**Keywords:** physical vein model, varicose vein, microfluidic, foam, foam sclerotherapy, physician compounded foam

## Abstract

Foam sclerotherapy is clinically employed to treat varicose veins. It involves intravenous injection of foamed surfactant agents causing endothelial wall damage and vessel shrinkage, leading to subsequent neovascularization. Foam production methods used clinically include manual techniques, such as the Double Syringe System (DSS) and Tessari (TSS) methods. Pre-clinical *in-vitro* studies are conducted to characterize the performance of sclerosing agents; however, the experimental models used often do not replicate physiologically relevant physical and biological conditions. In this study, physical vein models (PVMs) were developed and employed for the first time to characterize the flow behavior of sclerosing foams. PVMs were fabricated in polydimethylsiloxane (PDMS) by replica molding, and were designed to mimic qualitative geometrical characteristics of veins. Foam behavior was investigated as a function of different physical variables, namely (i) geometry of the vein model (i.e., physiological vs. varicose vein), (ii) foam production technique, and (iii) flow rate of a blood surrogate. The experimental set-up consisted of a PVM positioned on an inclined platform, a syringe pump to control the flow rate of a blood substitute, and a pressure transducer. The static pressure of the blood surrogate at the PVM inlet was measured upon foam administration. The recorded pressure-time curves were analyzed to quantify metrics of foam behavior, with a particular focus on foam expansion and degradation dynamics. Results showed that DSS and TSS foams had similar expansion rate in the physiological PVM, whilst DSS foam had lower expansion rate in the varicose PVM compared to TSS foam. The degradation rate of DSS foam was lower than TSS foam, in both model architectures. Moreover, the background flow rate had a significant effect on foam behavior, enhancing foam displacement rate in both types of PVM.

## Introduction

Venous incompetence in the lower limbs is a common disease. Varicose veins have long been considered a cosmetic problem but—if left untreated—they may lead to more advanced form of chronic venous dysfunction, such as chronic venous insufficiency (CVI) (Spiridon and Corduneanu, 2017). Varicose veins are generally treated in order to prevent venous stasis, reflux, hypertension, and ulceration (Gloviczki et al., 2011). Sclerotherapy is a minimally invasive technique for treating varicose veins, and involves the intravenous injection of a sclerosing solution to cause endothelial damage, vessel shrinkage, and subsequent neovascularization (Goldman et al., 2017). Foam sclerotherapy is a development of liquid sclerotherapy, where the sclerosing solution is mixed with a gas in order to produce a “microfoam” (bubble diameter: 100–500 μm) (Eckmann, 2009). The use of foamed sclerosants offers significant benefits, particularly in the treatment of larger lower-extremity veins (Hamel-Desnos et al., 2003; Ouvry et al., 2008; Smith, 2009). Notably, a limitation of liquid sclerotherapy is that the sclerosing agent mixes rapidly with blood and is “consumed” or de-activated by blood cells and plasma proteins, respectively (Parsi et al., 2008; Connor et al., 2015). On the other hand, a cohesive foam displaces blood away from the diseased vein, reducing the extent of sclerosant's deactivation and leading to a greater contact time with the endothelial layer. As a result, sclerosing foams can be more effective in damaging the vessel wall, and this is achieved at lower concentrations of active molecule compared to their liquid counterparts (Goldman et al., 2017).

The flow behavior of sclerosing foams is therefore an important determinant of their ability to fully prime the target vein, and to provide sufficient contact time with the endothelial layer to result in effective therapeutic outcomes. The ideal foam should be sufficiently viscous and have low bubble size dispersity, to result in adequate handling stability and cohesiveness upon injection (Star et al., 2018). Increasing the quantity of surfactant agent may result in greater foam stability; however, this is undesirable as higher concentrations of sclerosant may lead to increased risk of side effects (Peterson and Goldman, 2012). Phlebologists often generate foams manually; these types of foam are referred to as physician-compounded foams (or PCFs). The two most common techniques utilized for producing PCFs are (i) the double syringe system (DSS) and (ii) the Tessari (TSS) methods (Tessari et al., 2001). Both techniques involve mixing of a liquid sclerosant solution with a gas or gas mixture, which is achieved by passing the liquid and gas between two syringes joined together via a connector (Jia et al., 2007). The gas phase most commonly used is room air (RA); its high nitrogen (N_2_) content results in a foam that is more stable than N_2_-free PCFs. However, the low solubility of nitrogen in blood may be responsible for increased risk of gas embolism and neurological events. Other gases employed are clinical grade carbon dioxide (CO_2_), or CO_2_ and oxygen (O_2_) mixtures (Peterson and Goldman, 2011). Foams produced with these gases are reported to be less stable than RA-foams, due the high solubility of CO_2_ in blood, but the risk of embolism is largely reduced (Cavezzi and Tessari, 2009; Peterson and Goldman, 2011). However, they may be less effective in displacing blood, which could limit their therapeutic efficacy. The characteristics of PCFs can depend on many variables, including (i) the type of gas (Larmignat et al., 2008; Peterson and Goldman, 2011), (ii) the liquid:gas volume ratio (typically in the range 1:3–1:7), (iii) the type of surfactant (polidocanol or sodium tetradecyl sulfate) and its volumetric concentration (typically in the range 0.5–3%) (Hanwright et al., 2005; Van Deurzen et al., 2011), (iv) the type of connector used (straight connector in DSS and a 3-way valve in TSS) (Rao and Goldman, 2005), and (v) the number of passes between syringes (in the range 5–10) (Peterson and Goldman, 2011). This large parametric space often limits the possibility of comparing results from different studies; thus, there is a growing need to establish standardized methodological approaches to evaluate physical stability of PCFs (Hamel-Desnos et al., 2007).

Foam stability is often evaluated *in-vitro* by measuring macroscopic or microscopic parameters, such as foam half time (FHT), foam drainage time (FDT), bubble size distribution, and foam dwell time (FDT) (Kruglyakov et al., 2008; Carugo et al., 2016; Critello et al., 2017). In a typical experiment, a defined volume of foam is produced and delivered into a vessel, where changes to its physical properties are monitored as a function of time. FHT is the time required for half of the volume of sclerosing solution to revert to liquid (Nastasa et al., 2015). FDT is instead the time at which visible liquid drainage begins (Kruglyakov et al., 2008). Both parameters can be measured by observing drainage in a standing column of foam, and quantifying the height (or volume) of the liquid phase over time. This can be determined by analyzing photographic images of the foam column at increasing time points, or it can be inferred from changes in back-scattering or transmission of an incident light beam. These indicators of foam stability are however strongly dependent on the type and size of vessel in which the foam is contained (Carugo et al., 2015). Foam bubble size distribution can be measured by optical microscopy or light scattering techniques (Osei-Bonsu et al., 2015; Watkins and Oliver, 2017). The measured bubble size may however be strongly influenced by the invasiveness of the method used, and the time elapsed between foam production and analysis. A technique commonly used involves the injection of a foam sample between two glass plates, where foam containment in a small environment reduces the drainage and coarsening rates to facilitate imaging (Carugo et al., 2016).

The characterization methods reported above have been largely employed in the literature as a means to evaluate stability of sclerosing foams, and have been particularly useful for comparing different foam formulations (McAree et al., 2012; Cameron et al., 2013; Bai et al., 2018). However, the experimental systems used (i.e., syringes or vials) do not reflect dynamic conditions that are relevant to the end-point usage of the foam. Recently, Carugo et al. developed a model for the analysis of sclerosing foam behavior under more clinically relevant conditions. The model consisted of a 4 or 10 mm inner diameter polytetrafluoroethylene tubing, placed onto a platform with an adjustable inclination angle. Foam was injected into the tube, which was initially primed using a blood substitute, and its expansion/degradation rates were quantified using computational-based image analysis software. The model allowed to measure the foam dwell time, which is the time taken for a foam plug to recede over a unit distance (Carugo et al., 2015). It was however designed for usage under static fluidic conditions, and it did not replicate the varicose vein architecture.

In order to address these limitations of previous test methods, the work in this study aims to develop physical models replicating qualitative architectural characteristics of varicose veins and to employ them as a screening platform for comparing the flow behavior of different foam formulation methods. The developed biomimetic-inspired vein model (referred to as physical vein model, or PVM) allows recapitulating features of physiological and varicose veins, including circular cross-section, tortuous and swollen vessel morphologies, and physiologically relevant flow conditions. PVMs were employed to compare the flow performance of polidocanol-based PCFs, as a function of vessel geometry (straight vs. curved centerline), foam production technique (PCF vs. TSS), and volumetric flow rate. Moreover, it was demonstrated that models can be coated with endothelial cells, enabling future investigations of both mechanical and biological performance of sclerosing agents.

## Materials and Methods

### Physical Vein Models (PVM): Design and Manufacturing

**Figure 1:**
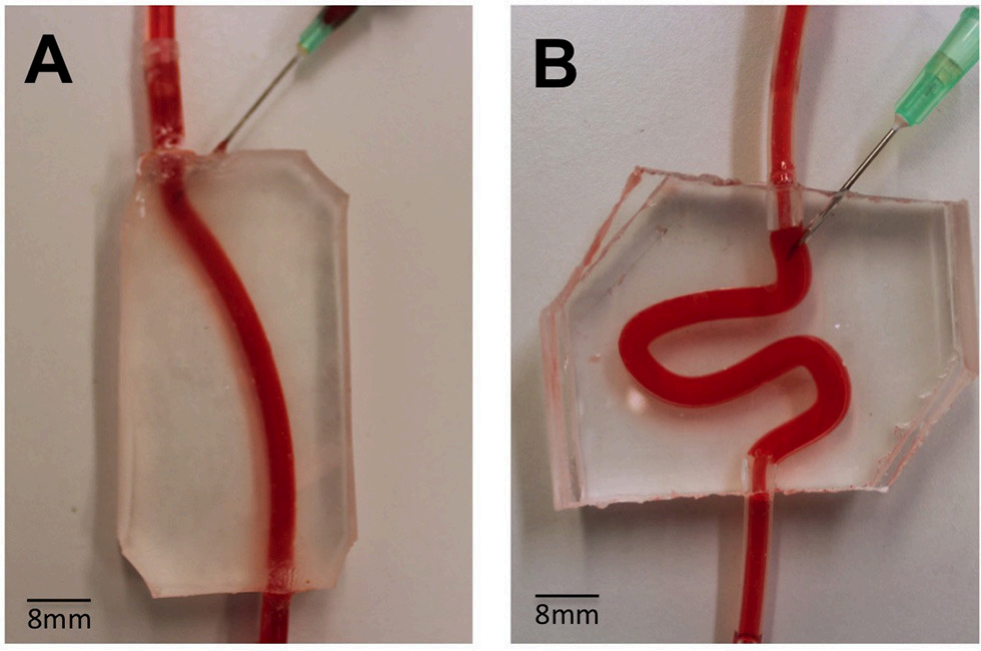
Assembled PVMs representing simplified physiological **(A)** and varicose **(B)** vein models, and demonstration of perfusion using a red dye. These models were employed to test the flow behavior of foams. The main channel was punctured with a needle (16G), in order to mimic the clinical process of injection more closely.

PVMs were fabricated via replica molding. Firstly, the 3D vein architecture was designed in SolidWorks (Dassault Systemes, SolidWorks Corporation, USA). Two different designs were generated to model both physiological and varicose veins. The physiological vein model comprised of a straight channel, whilst the varicose vein model comprised of a serpentine-like channel that replicated qualitatively a varicose vein geometry. In both models, channel length and inner diameter were set to 70 and 4 mm, respectively (Figure 1). The inner diameter replicated the average diameter of veins treated with sclerotherapy (Sandri et al., 1999), while the length was selected so that the model could accommodate at least 1 mL of foam. The volume of foam injected clinically depends on the size of the vessel segment to be treated (Chwała et al., 2015).

**Figure 2:**
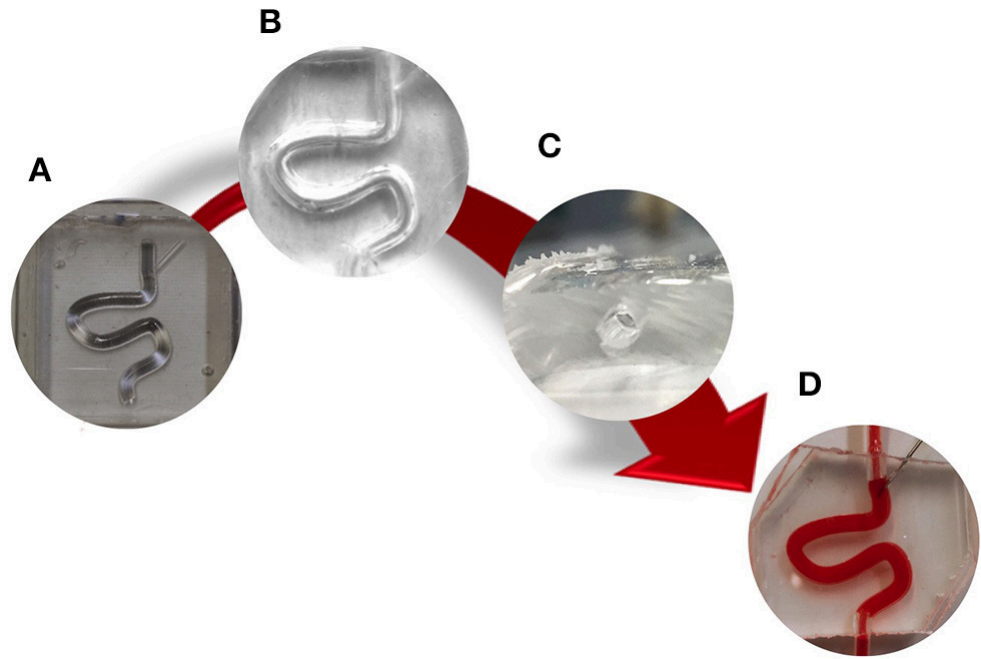
Schematic of PVMs production via replica molding. Firstly, **(A)** a CAD design of positive molds was generated and 3D printed. Secondly, **(B)** two PDMS layers were produced from the molds, aligned together, and permanently bonded via oxygen plasma treatment to obtain a fully circular channel cross-section **(C)**. Finally, the main channel was punctured with a 16G needle **(D)**.

The PVM manufacturing process is illustrated in Figure 2. Firstly, positive molds of the design were 3D printed using an Objet350 Connex printer (Haycroft Works, Buckholt Drive, UK). Two specular molds were fabricated, each with a semi-circular channel cross-section, in order to obtain a model with a fully circular cross-section by combining the two molds. In addition, alignment pins were added to the mold design in order to facilitate the alignment process. Polydimethylsiloxane (PDMS) prepolymer and curing agent (Sylgard® 184, Dow Corning Corporation, USA) were mixed at a weight ratio of 10:1 (w/w), and then poured onto the 3D-printed molds. PDMS was then cured in an oven, at 65°C for 1 h, and allowed to solidify. The two specular PDMS layers were then aligned together and permanently bonded via treatment with oxygen plasma (Tepla 300 Plasma Asher, PVA TePla AG, Germany) (Bodas and Khan-Malek, 2007). The main channel was punctured with a 16G needle (BD Biosciences, UK) in order to replicate the clinical foam administration process (Goldman et al., 2017). Inlet/outlet ports were connected with silicone tubing (4 mm outer diameter, Cole-Palmer, UK).

### Foam Production

PCFs were produced using polidocanol 1% (in buffered solution) and room air, at a liquid:gas volume ratio of 1:4. The foam production techniques employed were the double-syringe system (DSS) and Tessari (TSS) methods. In the DSS method, two syringes (10 and 5 mL, BD Biosciences, USA) were interconnected using a Combidyn™ adapter (B. Braun Melsungen, Germany), whereas in the TSS method they were connected via a three-way stopcock (Baxter, USA) (Rao and Goldman, 2005; Critello et al., 2017). In both techniques, the foam was produced by passing the polidocanol solution (liquid phase) from one syringe, 10 times into and out of the other syringe initially containing room air.

### Characterization of the Flow Behavior of Foams: Experimental Set-Up

**Figure 3:**
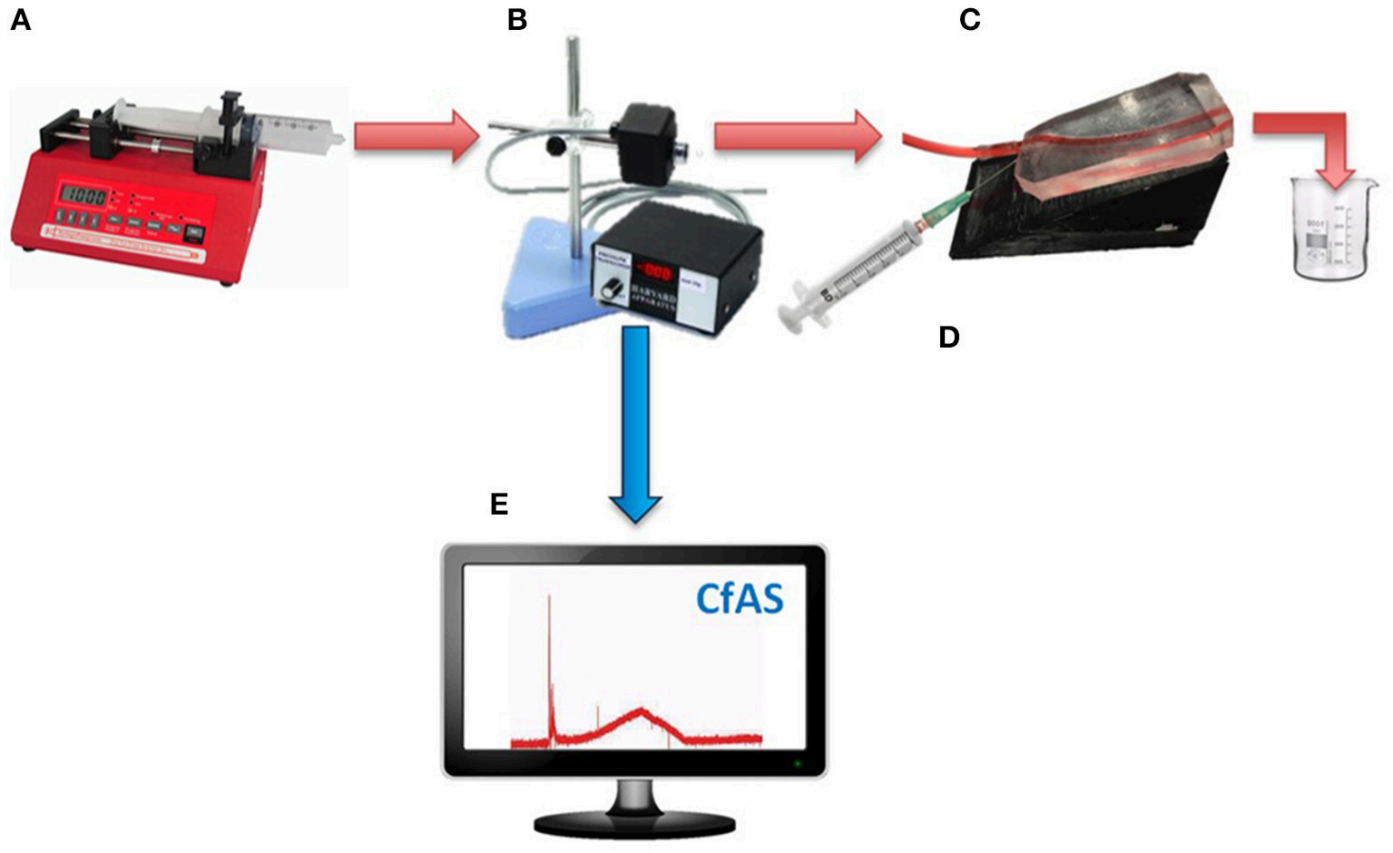
Schematic illustrating the set-up for evaluating the flow behavior of foams in the PVM model. A steady flow is imposed using a syringe pump **(A)**, and a pressure transducer **(B)** is positioned in line with the inlet tubing and prior to the PVM **(C)**. The foam is injected through the 16G needle into the main channel **(D)**. The pressure transducer is connected to a National Instruments I/O module. The NI-DAQ system supports analog and digital inputs, and communicates with the NI-DAQ software. A Matlab code is employed to store pressure data in an automated fashion **(E)**.

The experimental set-up consisted of a PVM lodged onto a 3D printed inclined platform (inclination angle of 25°), to replicate patient's leg elevation as in the clinical procedure. The inlet tube was connected to the PVM using a three-way stopcock. A blood substitute (30% v/v glycerol in purified water) with a fluid dynamic viscosity, μ, of 0.003 Pa × sec and density, ρ, of 1,078 kg/m^3^ (Pries et al., 1992) was conveyed through the vein model using a 10 mL syringe (BD Biosciences, USA). A steady flow of the blood substitute was imposed using a syringe pump (NE-1000 Programmable Single Syringe Pump, New Era Pump Systems, Inc., USA). A pressure transducer (Research Grade Blood Pressure Transducer, 230 VAC, 50 Hz, Harvard apparatus, UK) was positioned in line with the inlet tubing, and located 30 mm proximally to the PVM inlet (Figure 3). The pressure transducer was connected to a National Instruments I/O module (NI-DAQ, USB-6008, National Instrument, UK). The NI-DAQ system supports analog and digital inputs, and communicates with the NI-DAQ software (National Instrument, UK). A MATLAB® (The MathWorks Inc., USA) script was employed to store pressure data in an automated fashion.

### Characterization of the Flow Behavior of Foams: Experimental Procedures

As described earlier, the 10 mL syringe was filled with a blood substitute, which was conveyed through the PVM at constant flow rates. A clinically relevant volume of foam (1 mL) (Eckmann, 2009) was injected manually into the PVM through a needle, using a silicon-free plastic syringe with capacity of 5 mL. The static pressure of the blood surrogate at the PVM inlet was measured before, during and after injection of foam, for a fixed time of 100 s. The static pressure was set to 0 mmHg before injecting the foam. Results were transferred to a personal computer and analyzed as described in the following paragraphs. The volumetric flow rates investigated were 62.5, 72.0, and 125.0 mL/h (Figure 3). The corresponding inlet Reynolds number was calculated using Equation 1, where ρ is the density of the blood surrogate (kg/m^3^), *v* is the mean velocity of the blood surrogate (m/s), *D* is the hydraulic diameter of the vein model (m), and μ is the dynamic viscosity of the blood surrogate (Pa·s).

(1) $$\begin{array}{r} \text{Re}=\frac{\rho \nu D}{\mu }. \end{array}$$

The Reynolds number in these experiments ranged from 1.65 to 3.34, which is ~100–200 times lower than physiological values (Raju et al., 2004), in order to replicate quasi-static or impaired flow conditions occurring in diseased veins.

### Computational Foam Analysis System

**Figure 4:**
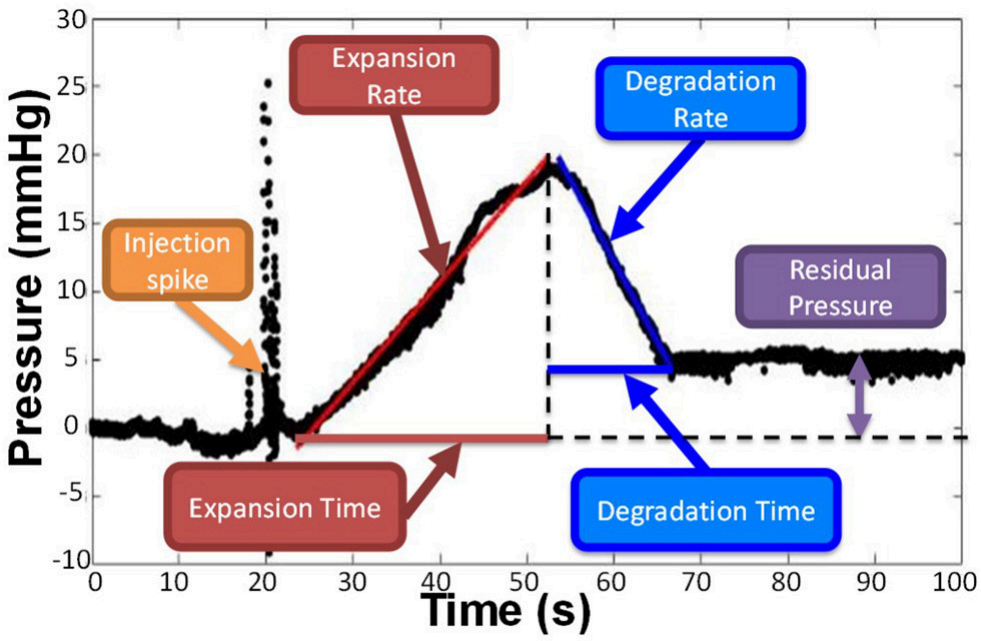
Representative pressure vs. time curve obtained by the CfAS. The plots were divided into three phases: (i) an increase of the pressure due to the expansion of the foam inside the channel, (ii) a decrease of the pressure caused by the degradation of the foam, and (iii) an initial peak due to the injection.

A computational foam analysis system (CfAS) was developed with the aim of analyzing the pressure recordings upon foam injection. The analysis system was designed using MATLAB R2016a software (The MathWorks Inc., USA) with a flexible user-intended interface. The software read the experimental pressure measurements and performed a sequence of semi-automated operations, allowing extraction of relevant parameters from the pressure-time data. The pressure-time curve could be divided into three phases (Figure 4): (i) an initial spike due to the foam injection procedure, (ii) an almost linear increase in pressure due to the expansion of the foam within the PVM, and (iii) an almost linear decrease in pressure caused by foam degradation and “washing out.” The CfAS calculated the slope of phases (ii) and (iii), which were referred to as expansion rate (ER) and degradation rate (DR), respectively. In addition, expansion time (ET) and degradation time (DT) were also quantified.

A representative pressure profile is illustrated in Figure 4, from which four different phases can be identified:

- *Foam injection*. This phase was associated with a rapid pressure spike, likely due to the insertion of the needle within the PVM or other mechanical perturbations associated with the injection procedure. Peak pressure values in this phase ranged from 10 to 50 mmHg.
- *Foam plug expansion*. While the foam plug expanded within the PVM, the backpressure increased almost linearly. This is due to the significantly higher effective viscosity of foams compared to the blood surrogate, as reported in previous studies (Wong et al., 2015), leading to increased hydraulic resistance. The linear pressure increase is coherent with previous studies investigating foam behavior in a tube model, which revealed an almost linear increase in foam plug length during expansion (Carugo et al., 2015). The CfAS allowed quantifying the slope of the plug expansion phase, which was herein referred to as expansion rate (ER). It is hypothesized that more cohesive foams would fractionate more slowly and dilute less rapidly with the blood substitute, thus resulting in lower ER and higher peak pressures. The expansion time (ET) was also calculated from the pressure-time curve; higher ET corresponds to a longer contact time between the foam and the inner surface of the PVM.
- *Foam plug degradation*. Once expansion was complete, the foam plug underwent degradation. This is due to a combination of processes, including foam drainage, coarsening, and the “washing out” action exerted by the background flow of a blood substitute. Foam degradation resulted in a reduction in hydraulic resistance, leading to an almost linear drop in PVM backpressure. As for the degradation phase, the shape of the pressure profile during degradation is consistent with prior studies (Carugo et al., 2015). The CfAS allowed quantifying the slope of the plug degradation phase, which was referred to as degradation rate (DR). It is hypothesized that more cohesive foams would destabilize more slowly, thus resulting in lower DR. The degradation time (DT) was also calculated from the pressure-time curve; higher DT corresponds to a longer contact time between the foam and the inner surface of the PVM.
- *Residual pressure*. The pressure level at the end of the degradation phase could be equal or greater than the initial pressure (i.e., at the time of injection). Because of gravitational separation, bubbles accumulated at the top surface of the vein model. If sufficiently stable, they would remain in place for the duration of the pressure recording (up to 100 s), causing the residual pressure to be greater than the initial pressure.

### Statistical Analysis

Comparisons between foam production methods were performed using an unpaired Student's *t*-test for two groups analysis, or one-way ANOVA in the case of more than two groups. Statistical significance was assumed for *p* < 0.05. All statistical tests were performed with Prism software (GraphPad Software Inc., USA). All data were reported as the mean ± SD of at least six independent repeats of the same experiment.

### Cell Seeding

HUVECs (human umbilical venous endothelial cells) were seeded in the PVM models. HUVECs were extracted from umbilical cords, and this was carried out in accordance with the Human Tissue Act (2004) and the recommendations of Southampton & South West Hampshire Research Ethics Committee B with Governance provided by the University of Southampton Research Governance Office. Umbilical cords were collected from the Princess Anne Hospital (Southampton, UK) from non-complicated natural vaginal births following agreed ethical collection protocols [Local Research Ethical Committee (LREC); Ref: 07/H0502/83]. Firstly, the PDMS devices were washed four times with 70% ethanol for sterilization. Then, HBSS (Hanks' Balanced Salt Solution, Thermo Fisher Scientific, USA) was conveyed through the channels to remove ethanol traces. Afterwards, the inner surfaces of the device were coated with 3 mL of different proteins: 50 μl/mL of rat type I collagen (100 μg/mL; Gibco™, UK) or (ii) 100 μl/mL of fibronectin (Sigma, UK). The device was subsequently placed at 37°C in a 5 % CO_2_ incubator for 2 h, followed by rinsing with HM (HUVEC Medium, Thermo Fisher Scientific Inc., USA).

The HUVECs suspension was injected into the proteins-coated channels (at a concentration of 4–5 × 10^6^ cells/mL), and the device was incubated for 2 h. In order to achieve complete coating, the device was then turned upside down, and primed with a fresh HUVEC suspension. The device was finally incubated overnight to promote cell attachment.

### Image Acquisition

Bright field images of HUVECs within the PVM models were acquired with an optical microscope (Olympus, CKX41, Japan). Images were taken of live samples every 24–48 h.

## Results

### Characterization of the Flow Behavior of Foams in PVM Models

The PVM models developed in this study have been employed to characterize the flow behavior of sclerosing foams, by measuring static pressure of a blood surrogate at the PVM inlet.

**Figure 5:**
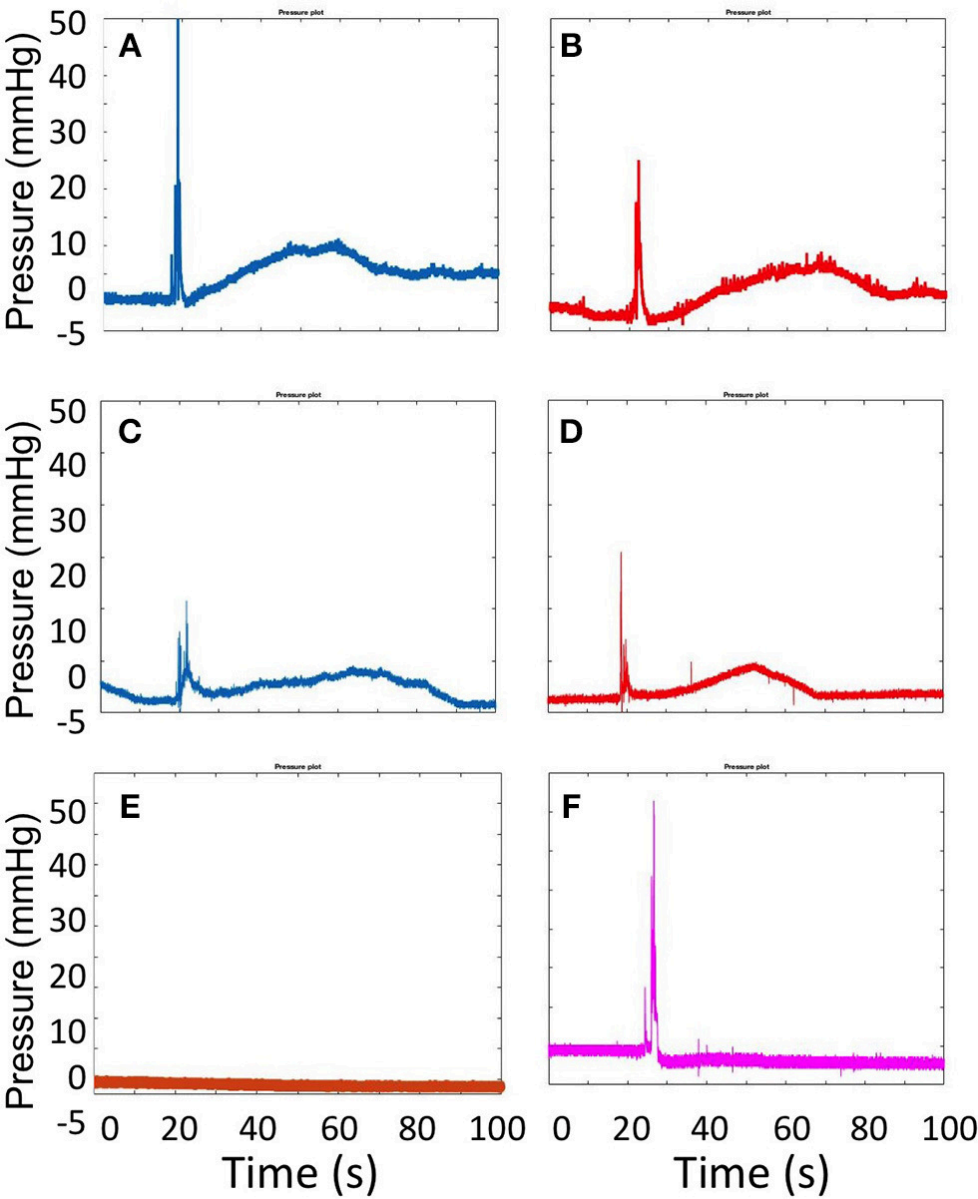
Pressure plots obtained from the CfAS while injecting DSS **(A,C)** and TSS **(B,D)** foams. The upper panels show the recordings obtained using the straight channel geometry, whereas the lower panels show the recording obtained using the serpentine-like channel geometry. As a control test **(E)**, the pressure was measured without injecting foam, at a background constant flow rate (72 mL/h). The pressure inside the vein models was also measured while injecting the foam in the absence of a blood-surrogate flow **(F)**.

Representative pressure-time curves obtained by CfAS, for both DSS (blue line) and TSS (red line) foams are shown in Figure 5. Both PVM geometries, i.e., straight (Figures 5A,B) and serpentine-like (Figures 5C,D), showed similar pressure profiles containing the four phases discussed earlier. As a control test, pressure was measured without injecting the foam, at a constant background flow rate of 72 mL/h (Figure 5E). As expected, no pressure variation was detected in these experiments for both PVM geometries. In addition, tests were also performed where foams were injected in the absence of background flow (Figure 5F). In this case, only the pressure spike corresponding to foam injection was present, due to the absence of a background pressure-driven flow.

**Figure 6:**
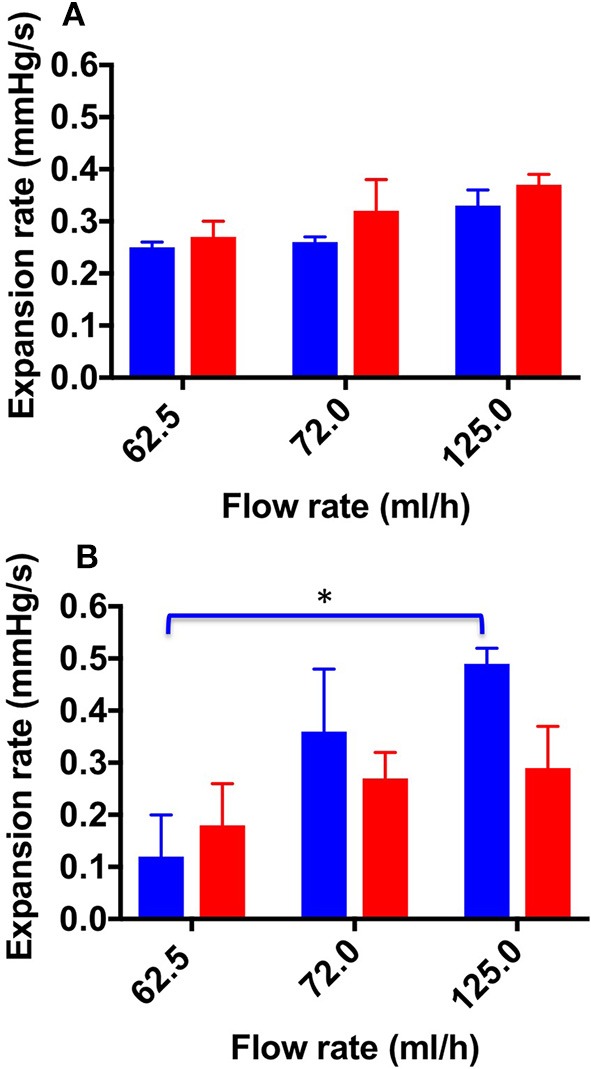
Expansion rate values at the different flow rates investigated, and different foam production methods (TSS blue bars, DSS red bars). Measurements were obtained from the straight **(A)** and curved **(B)** channel geometry. Data represent the average of 6 measurements ± SD. An asterisk (^*^) indicates that differences between mean values are statistically significant (*p* < 0.05). The experiment was repeated six times, and results are reported as mean value ± standard deviation.

Figure 6 shows the ER values determined for both TSS and DSS foams at all flow rates investigated, using both physiological (Figure 6A) and varicose (Figure 6B) PVMs. Lower ER is herein regarded as a therapeutically favorable property of foams, as it is indicative of higher foam cohesion and longer persistence in the vein. In the physiological PVM, TSS and DSS had comparable ER at all flow rates investigated. Values were equal to 0.25 ± 0.01 mmHg/s (62.5 mL/h), 0.26 ± 0.01 mmHg/s (72.0 mL/h), and 0.33 ± 0.04 mmHg/s (125.0 mL/h) for TSS; and 0.27 ± 0.03 mmHg/s (62.5 mL/h), 0.32 ± 0.06 mmHg/s (72.0 mL/h), and 0.37 ± 0.02 mmHg/s (125.0 mL/h) for DSS. On the other hand, in the varicose PVM model, DSS foam had lower ER compared to TSS, particularly at the highest flow rate (0.49 ± 0.03 mmHg/s for TSS and 0.29 ± 0.08 mmHg/s for DSS). Differences in foam behavior may be attributed to their bubble size distribution and drainage kinetics (Carugo et al., 2015).

A one-way ANOVA was conducted to evaluate whether ER of both types of foam depended on the inlet flow rate. With respect to the TSS group, a significant difference was observed between all flow rates investigated; indeed, the average ER value increased with increasing the inlet flow rate.

**Figure 7:**
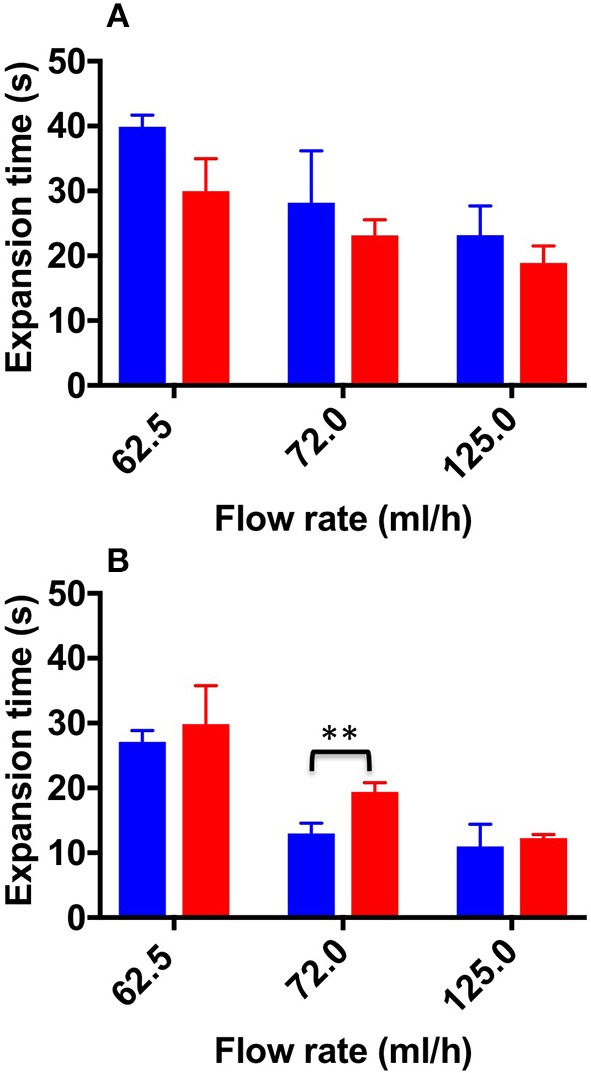
Expansion time values at the different flow rates investigated, and different foam production methods (TSS blue bars, DSS red bars). Measurements were obtained from the straight **(A)** and curved **(B)** channel geometry. Two asterisks (^**^) indicate that differences between mean values are very statistically significant (*p* < 0.01). The experiment was repeated six times, and results are reported as mean value ± standard deviation.

**Figure 8:**
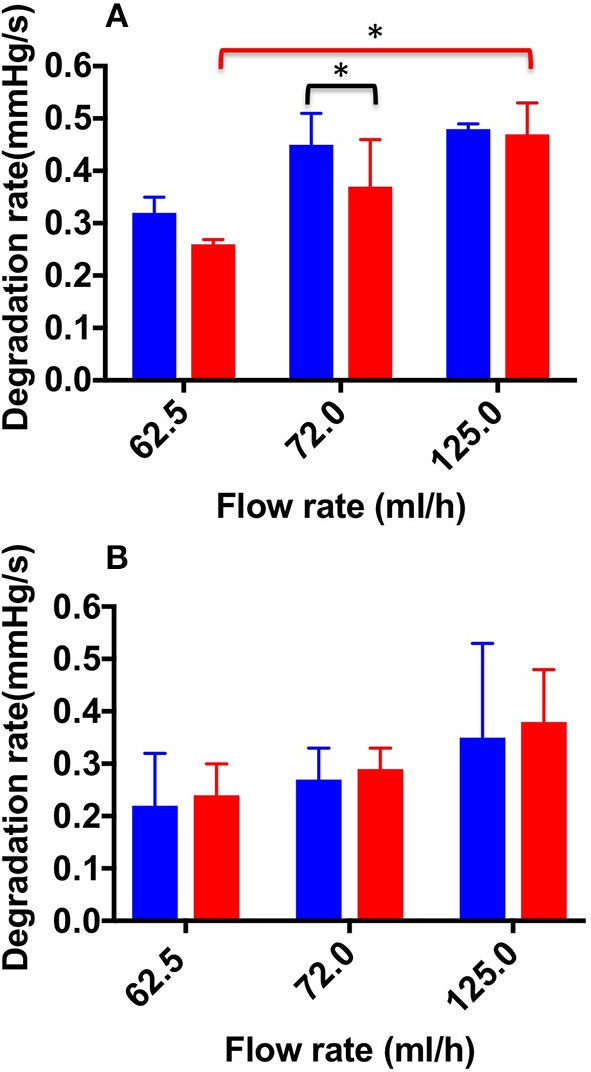
Degradation rate values at the different flow rates investigated, and different foam production methods (TSS blue bars, DSS red bars). Measurements were obtained from the straight **(A)** and curved **(B)** channel geometry. An asterisk (^*^) indicates that differences between mean values are statistically significant (*p* < 0.05). The experiment was repeated six times, and results are reported as mean value ± standard deviation.

Figure 7 shows the ET values determined for both TSS and DSS foams, at all flow rates investigated. As expected, the expansion time (ET) was slightly higher for TSS at 62.5 and 72.0 mL/h (39.9 ± 1.00 and 28 ± 8.00 s, respectively) compared to DSS (29.9 ± 5.00 and 23 ± 2.00 s, respectively). However, in the physiological PVM no statistical difference was determined between PCFs. In the varicose PVM, TSS foam had lower ET compared to DSS foam at all flow rates investigated, consistently with the expansion rate data reported previously. Statistical difference between TSS and DSS was found at 72.0 mL/h (12.98 ± 1.60 s and 19.35 ± 1.430 s for TSS and DSS, respectively) (Figure 8B). In both PVM models, increasing the background flow caused a reduction of ET, likely due to a “washing out” effect of the blood surrogate, as discussed earlier. In the varicose vein model, DSS foam demonstrated greater ability to oppose this effect, resulting in higher contact time with the vessel wall.

Figure 8 shows DR values determined for both TSS and DSS foams, at all flow rates investigated. In the physiological PVM, at the lowest flow rate (62.5 mL/h), DSS foam had a lower DR (0.26 ± 0.01 mmHg/s) compared to TSS foam (0.32 ± 0.03 mmHg/s). Significant difference in DR between TSS (0.45 ± 0.06 mmHg/s) and DSS (0.37 ± 0.09 mmHg/s) foams was found at 72.0 mL/h. At the highest flow rate (125.0 mL/h) both types of foam had similar DR (0.48 ± 0.03 mmHg/s for TSS and 0.47 ± 0.07 mmHg/s for DSS), suggesting that foam degradation performance is dominated by the background flow at these higher flow rates. A one-way ANOVA was performed to evaluate the effect of background flow rate on DR, for both types of PCF. With respect to the DSS group, a significant difference was observed with increasing the inlet flow rate; indeed, the average DR value increased with increasing the flow rate. With respect to the TSS group, no significant difference was found by varying the flow rate. Both foams had comparable degradation performance across the two PVM geometries, suggesting that once a foam plug has been established into the vein, its degradation dynamics is not significantly affected by the vessel architecture.

**Figure 9:**
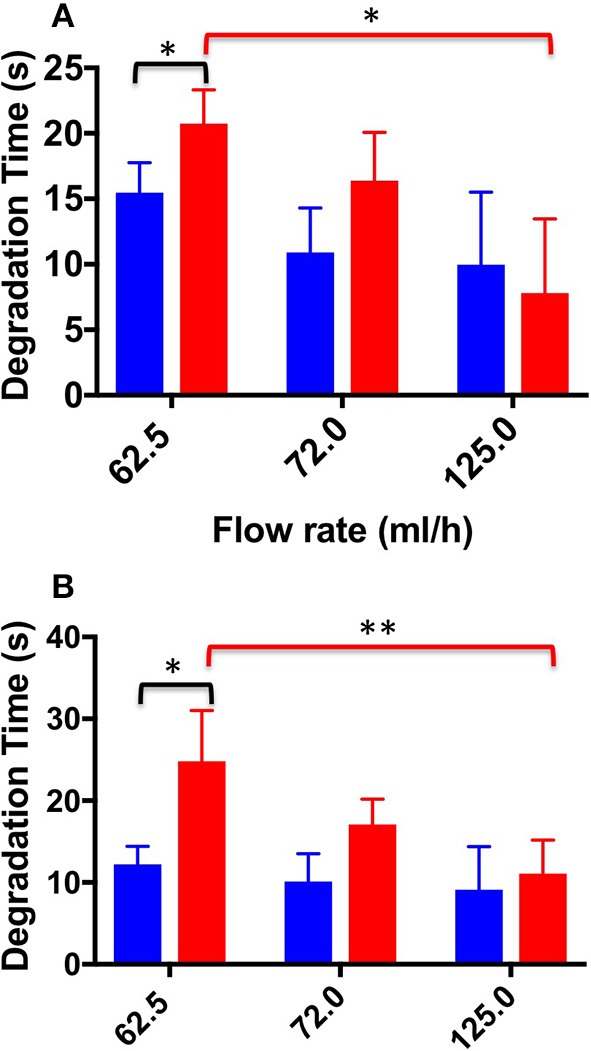
Degradation time values at the different flow rates investigated, and different foam production methods (TSS blue bars, DSS red bars). Measurements were obtained from the straight **(A)** and curved **(B)** channel geometry. An asterisk (^*^) indicates that differences between mean values are statistically significant (*p* < 0.05) and two asterisks (^**^) indicate that differences between mean values are very statistically significant (*p* < 0.01). The experiment was repeated six times, and results are reported as mean value ± standard deviation.

Figure 9 shows DT values determined for both TSS and DSS foams at all flow rates investigated. In the physiological PVM model, at the lower flow rate (62.5 mL/h), DSS foam had a statistically higher DT (20.73 ± 2.60 s) compared to TSS foam (15.46 ± 2.30 s). At the intermediate flow rate, the average DT value was still higher compared to TSS (16.38 ± 3.70 s and 10.90 ± 3.40 s, respectively). At the highest flow rate (125.0 mL/h) both PCFs had comparable DT (9.97 ± 5 s for TSS and 7.8 ± 5.6 s for DSS). As expected, foams with lower degradation rate had a longer degradation time. Similar observations were made using the varicose PVM model, where at the lowest flow rate (62.5 mL/h) DSS foam had statistically higher DT (24.8 ± 6.20 s) compared to TSS. Differences between foam types reduced with increasing the inlet flow rate. At 125.0 mL/h, both types of foam presented similar DT (9.1 ± 5.3 s for TSS and 11.07 ± 4.1 s for DSS). A one-way ANOVA was conducted to evaluate the effect of flow rate on DT for both types of foam. With respect to the DSS group, results show that there is a significant difference between DTs measured at increasing flow rates, for both types of geometry. Moreover, DT was not significantly influenced by the PVM geometry, as for the degradation rate. With respect to the TSS group, no significant difference was found by varying the background flow rate.

### Cell Seeding and Channel Functionalization

**Figure 10:**
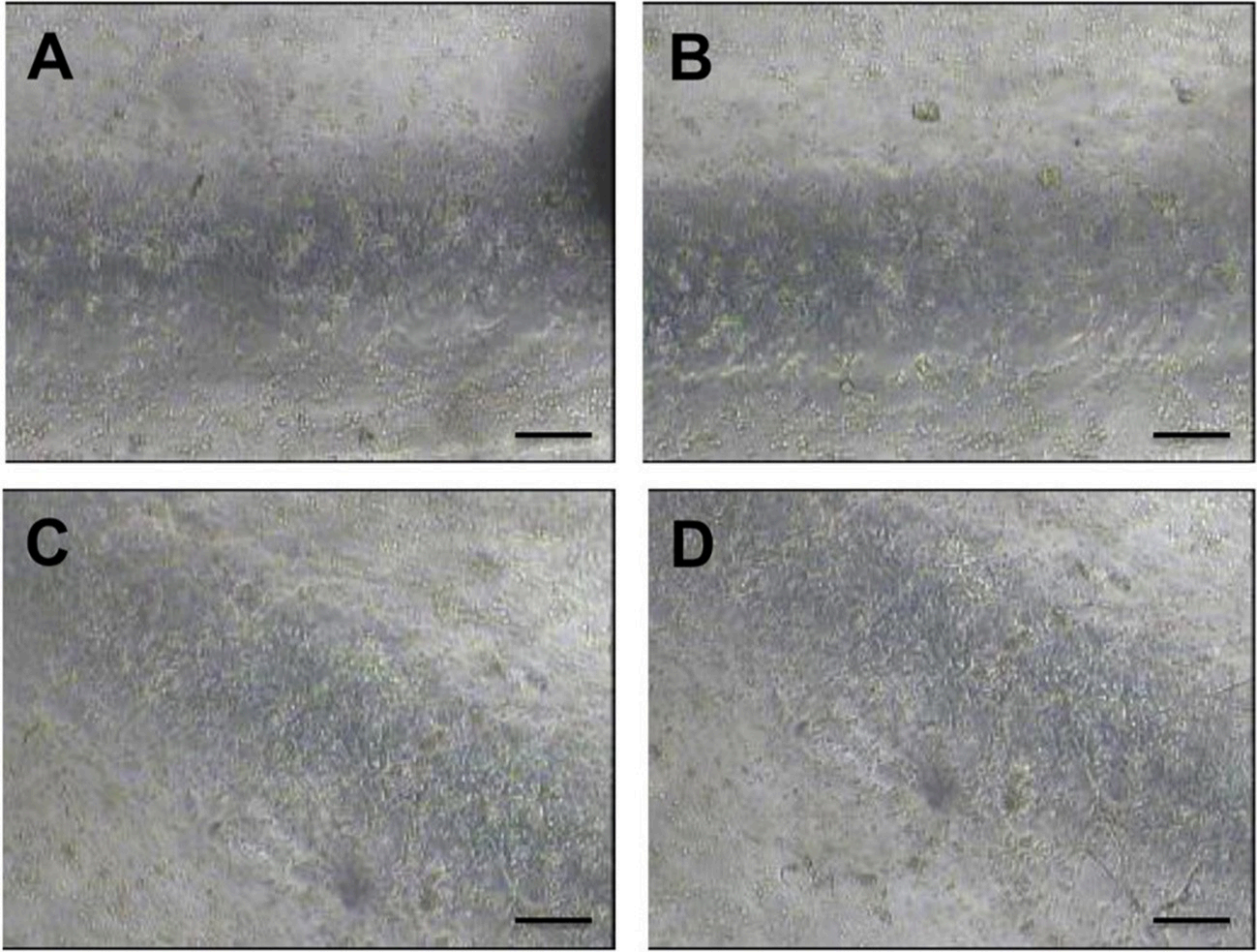
Bright field microscope images of HUVECs cultured within the fully circular PVM channels. Images on the left show the lower channel wall coated with collagen **(A)** and fibronectin **(C)**, respectively. Images on the right show the upper channel wall coated with collagen **(B)** and fibronectin **(D)**, respectively. Images (4x magnification) were taken after 48 h from cell seeding. Scale bars are 200 μm long.

With the aim of developing PVM models that can be coated with an endothelial monolayer, seeding of HUVECs over the inner PDMS surfaces of the device was investigated. PDMS has been extensively used for cell culture in microfluidic devices (Choi et al., 2013), because of its optical transparency, low cost, gas permeability, and biocompatibility. However, it is not an ideal substrate surface for cell attachment, due to its hydrophobicity. As shown in Figure 10, HUVECs attached and uniformly distributed over the surface of both lower and upper walls of the circular channels, upon coating with extracellular proteins. Particularly, fibronectin coating resulted in more effective seeding, with a larger surface area covered by HUVECs. It is important to highlight that PVM devices have a larger channel (4 mm in diameter) compared to microchannels typically used in vasculature-on-a-chip devices (10–400 μm) (Tien, 2014). This makes the cell coating process more challenging, given the larger surface area to be covered. However, despite channels were larger in this study, it was possible to obtain a relatively homogenous coating using a lower cell seeding concentration compared to previous studies (typically in the range 5 × 10^5^−1.25 × 10^7^ cells/mL) (Chung et al., 2009; Kim et al., 2013).

## Discussion

In this study, a novel experimental method to quantify and compare the flow behavior of sclerosing foams was developed. The method provided a quantitative determination of fluid pressure upon foam administration, within models of either physiological or varicose veins (referred to as physical vein model, or PVM).

When a cohesive foam is injected into the PVM, it forms a plug that displaces the blood substitute. The foam plug however degrades over time, due to its intrinsic instability and the “washing out” action of the background flow. Using our model system, we were able to characterize these phenomenological behaviors for the first time, by measuring the static pressure of a blood surrogate at the PVM inlet (Figure 5).

It is well-known that sclerosing foams produced using different techniques differ in their “static” physical properties. In this study, we evaluated for the first time the dynamic flow behavior of sclerosing foams, by analyzing their expansion and degradation within qualitative models of both physiological and varicose veins. In particular, the behavior of different PCFs was compared, at varying volumetric flow rates (in the range 62.5−125.0 mL/h).

Overall, the results reported in this study show that TSS and DSS foams had comparable expansion rate in the physiological vein model, whereas TSS had faster expansion rate in the varicose model (Figures 6, 7). Therefore, DSS foam has the ability to expand more slowly within a varicose vein model architecture, resulting in longer contact time (ET) with the vein wall upon injection. These results are consistent with prior studies showing that DSS foams are more cohesive than TSS foams in a tube model, in the absence of a background flow rate (Carugo et al., 2015).

Results also demonstrated that the flow field within the target vein can significantly influence the expansion dynamics of sclerosing foams. DSS foam was slightly less sensitive to changes in the background flow rate, suggesting that more cohesive foams may offer higher resistance to the “washing out” effect of the blood flow during expansion. Reducing blood flow rate during administration (i.e., via vein compression) may thus be preferable to enhance therapeutic efficacy.

With respect to the degradation dynamics of PCFs (Figures 8, 9), at the lowest flow rate investigated DSS foam had lower degradation rate compared to TSS foam. This was likely due to the slower coarsening and drainage rate of DSS foams, coherently with previous studies (Carugo et al., 2015). Increasing the inlet flow rate resulted in PCFs having comparable degradation rate, suggesting that foam degradation performance is dominated by the background flow in these conditions. Interestingly, there was no significant difference in the degradation dynamics between the two PVM geometries investigated; suggesting that once a foam plug has been established into the vein, its degradation dynamics is not significantly affected by the vessel architecture.

It is important to highlight that expansion and degradation dynamics taken at the lowest flow rate are likely to be more representative of the flow conditions in a diseased (i.e., varicose) vein. In these conditions, DSS presented a slightly superior performance compared to TSS.

Finally, it was demonstrated that PVM models can be lined with endothelial cells in order to recreate the endothelial layer (Figure 10). The degree of endothelial damage upon treatment with foam can be employed as an indicator of therapeutic efficacy. In future work, we will employ these cell-coated PVM models to investigate the biological effects of sclerosing foams and correlate them with foam mechanical behavior.

## Conclusion

In this study, we described the development of physical vein models replicating the qualitative architecture of physiological and varicose veins, and their utility as model platforms to screen the flow behavior of sclerosing foams, upon different formulation and administration conditions.

A simple method to manufacture vein models was developed, which aimed at generating channels with circular section and with a geometry that recapitulates some characteristics of the varicose vein. An experimental protocol was also established to investigate the flow performance of foams at conditions relevant to their clinical administration. Notably, the experimental set-up replicated some aspects of the clinical process of foam injection, including the use of a needle, patient's leg elevation, and the presence of a background blood flow.

Fluid pressure at the PVM inlet was measured during foam administration, which revealed different phases of the foam expansion and degradation dynamics. Particular emphasis was given to expansion and degradation of the foam plug, as indicators of its therapeutic efficacy.

As reported in previous studies (Carugo et al., 2015), the cohesiveness of foams is highly dependent on their rheological properties, which in turn are influenced by the bubble size distribution and foam drainage kinetics. Previous results showed that foam produced using the DSS method were more stable and presented longer dwell time compared to TSS foams (Carugo et al., 2015). Consistently with these previous observations, in our dynamic study DSS foam had longer degradation time and slower degradation rate than TSS foam. With respect to the expansion dynamics, no significant difference between the two foam formulations was found in the physiological vein model, whereas DSS had slower expansion in the varicose model. Differences in foam behavior across different model geometries could be attributed to the broader bubble size distribution of TSS foam compared to DSS foam; although these aspects merit further investigation.

In conclusion, the physical vein models and experimental methods developed in this study provide a novel technology platform to measure the behavior of different formulations of sclerosing foams, at physical conditions that resemble their clinical administration. They could therefore be employed as an additional test method in the pre-clinical pipeline, to innovate foam formulation and administration procedures. Moreover, we demonstrated that PVM models are suitable for coating with endothelial cells, which enables future investigations to correlate flow performance of sclerosing agents with their biological effects.

It should be noted that the PVM models reported in this study do not replicate the presence of venous valves, branching structures, or the mechanical properties of the vein wall, which may affect the flow behavior of foams. Ongoing research is focusing on the incorporation of these additional architectural and functional characteristics. Moreover, a more faithful replication of the physiological boundary conditions (including changes due to clinical practices; i.e., vein compression) will be considered in the future.

## Author Contributions

EB conducted all experiments and data analyses, designed all experimental procedures, and wrote the manuscript. DC co-designed all experimental procedures, wrote the Matlab code, co-wrote the manuscript and supervised the project. AL, SJ, and VP contributed to the design and implementation of the research and to the analysis of the results. All the other authors helped supervise the project.

### Conflict of Interest Statement

EB is in receipt of a Doctoral Training Partnership funded from EPSRC and Biocompatibles UK Ltd, a BTG International group company. AL, SJ, and VP are paid employees of Biocompatible UK Ltd. The remaining authors declare that the research was conducted in the absence of any commercial or financial relationships that could be construed as a potential conflict of interest.
